# Association Between Sleep Duration, Obesity, and School Failure Among Adolescents

**DOI:** 10.1177/1059840520901335

**Published:** 2020-01-24

**Authors:** Sofie Litsfeldt, Teresa M. Ward, Peter Hagell, Pernilla Garmy

**Affiliations:** 1 Faculty of Health Sciences, Kristianstad University, Kristianstad, Sweden; 2 Department of Psychosocial and Community Health, School of Nursing, 7284 University of Washington, Seattle, WA, USA; 3 Department of Health Sciences, Clinical Health Promotion Centre, Lund University, Lund, Sweden

**Keywords:** sleep, obesity, school success, adolescents, cross-sectional

## Abstract

The aim of this study was to investigate the association between sleep duration, overweight/obesity, and school failure using data obtained from self-reported questionnaires completed by 13- to 15-year-olds in Sweden (*n* = 1,363; 50.7% female). The height and weight of the participants were measured by school nurses. A multiple logistic regression analysis was used to analyze the association between sleep duration and overweight/obesity, school failure, and perceived economic situation. A short sleep duration (<7 hr) was associated with overweight/obesity (*p* = .001), school failure (*p* = .007), and poorer perceived economic situation (*p* = .004). Modifying the sleep habits of adolescents is a potential target for obesity intervention as well as for improving school success. This information is particularly well suited for school nurses to disseminate to students and their parents.

Globally, children and adolescents have been sleeping less over the past century (Basch et al., 2014; Matricciani et al., 2012). Adolescents generally require 8–10 hr of sleep each night (Hirshkowitz et al., 2015), but epidemiological studies in the United States have shown that a third of 12- to 14-year-olds and over half of 15- to 17-year-olds sleep fewer than 7 hr on an average school night (Basch et al., 2014; Taveras et al., 2008). Only 10% of 15- to 17-year-olds and 29% of 12- to 14-year-olds sleep more than 9 hr on an average school night (Hirshkowitz et al., 2015). Fuligni et al. (2018) argue that optimal sleep duration differs for different developmental outcomes among adolescents. For example, peak levels for mental health were found among adolescents who slept 8.75–9.00 hr per night, but the limits that affected academic success were 7.00–7.50 hr. Sleep is important for memory consolidation and creativity, and sleep deprivation causes learning problems, including problems with concentration and discipline (Kryger et al., 2011).

Obesity in adolescents is a public health concern, and its prevalence has increased globally (World Health Organization, 2017). In Swedish 14-year-olds, 22% of boys and 20% of girls are overweight, with 5% of boys and 4% of girls considered obese (Eriksson et al., 2018). Obesity and overweight in childhood and adolescence can lead to several health complications such as diabetes, cardiovascular disease, obstructive sleep apnea, a higher risk of obesity in adulthood (Silva et al., 2011; Taveras et al., 2008; Yanovski, 2015), and psychosocial problems such as depression (Daniels, 2009a).

The relationship between sleep duration and body weight is complex, but prior studies suggest both biological and behavioral involvement (Miller et al., 2015). Biologically, short sleep duration is associated with changes in metabolic hormones such as reduced leptin and increased ghrelin levels (Boeke et al., 2014; Leproult & Van Cauter, 2010). Changes in the levels of these hormones are associated with increased appetite, which in turn may lead to overeating and body mass index (BMI) increases (Hirotsu et al., 2015). Two prior meta-analyses have shown that short sleep duration is a risk factor for obesity in children and adolescents worldwide (Fatima et al., 2016; Felso et al., 2017), but the definition of short sleep was inconsistent and not always reported. Recent studies from North America (United States and Puerto Rico) and Asia (South Korea) have shown an association between sleeping <7 hr per night and high BMI in adolescents (Grandner et al., 2015; Koinis-Mitchell et al., 2017; Lee et al., 2016). However, a large study from the United States (Calamaro et al., 2010) failed to find an association between <6 hr sleep per night and obesity in adolescents. Rather, environmental factors such as watching television (TV) for more than 2 hr per day and depression were associated with obesity (Calamaro et al., 2010). The etiology of adolescent obesity is complex and characterized by several contributing factors (Lobstein et al., 2015), including psychosocial stress, poor eating habits, and reduced physical activity (Kumar & Kelly, 2017) as well as being socioeconomically disadvantaged (Buzek et al., 2019; Kelly et al., 2016).

## Aim

This study aimed to investigate the associations between sleep duration, overweight/obesity, and school success among 13- to 15-year-old adolescents.

## Method

This study is part of a larger project called “Sleep and lifestyle in school age children” (Trial Registry ISRCTN 17006300, BioMed Central, 2017). The study was approved by the Regional Ethical Review Board in Lund, Sweden. Prior to data collection, a letter containing information about the study was sent to the adolescents and their guardians to invite them to participate, and written informed consent was obtained. All procedures were conducted in accordance with the Declaration of Helsinki.

### Sample and Data Collection

The sample consisted of students in Grades 7 and 8 (13–15 years old) in four municipalities in southern Sweden, with the data collected during the 2015–2017 academic years. Questionnaires were distributed to the school nurses in the included municipalities, and the participating students completed the questionnaires at individual routine appointments with the nurses during school hours.

### Data Measures

The self-reported questionnaire used here has earlier been psychometrically tested and found to be valid and reliable for this age-group (Garmy et al., 2012). There is evidence supporting the validity of self-reported sleep from a study with 14- to 18-year-old adolescents that showed a high correlation between their self-reported sleep duration and objectively measured sleep duration using actigraphy (Wolfson et al., 2003). The questionnaire included the following questions: “When I have school the next day, I usually sleep around…” (hours and minutes), “How financially well off do you think your family is?,” with five response options: “very well off,” “rather well off,” “average,” “not that good,” and “not good at all.” In Table 1, the response options “very well off” and “rather well off” are grouped as a “good” economic situation, whereas the responses “not good” and “not good at all” are grouped as a “poor” economic situation. In Table 2, the responses are dichotomized, and “very well off” and “rather well off” were coded 0 and all other options were coded 1.

**Table 1.** Association Between Short Sleep Duration (<7 hr) and Sex, Age, Economic Situation, School Failure, and Overweight/ Obesity Among Students Aged 13–15.

	Sleep <7 hr	Sleep ≥7 hr	χ^2^ (*df*)	*p* Value
	*n* = 117 (8.4%)	*n* = 1,246 (89.8%)		
	*n* (%)	*n* (%)		
Sex				
Girl	67 (9.6)	630 (90.4)	1.92 (1)	.176
Boy	50 (7.5)	616 (92.5)		
Age				
13 Years	5 (3.0)	164 (97.0)	8.6 (2)	**.014**
14 Years	110 (9.3)	1,072 (90.7)		
15 Years	2 (16.7)	10 (83.3)		
Perceived economic situation^a^				
Good	85 (7.6)	1,028 (92.4)	12.59 (2)	**.002**
Average	21 (11.7)	158 (88.3)		
Poor	6 (26.1)	17 (73.9)		
School failure^b^				
No failed subjects	89 (7.6)	1,081 (92.4)	14.86 (2)	**.001**
1–2 Failed subjects	18 (12.9)	121 (87.1)		
≥3 Failed subjects	10 (21.7)	36 (78.3)		
BMI				
Normal weight	89 (7.7)	1,071 (92.3)	13.0 (2)	**.001**
Overweight	18 (11.5)	139 (88.5)		
Obese	10 (21.7)	36 (78.3)		

*Note*. *n* = 1,363. χ^2^ = Pearson’s chi-square; *df* = degrees of freedom; BMI = body mass index. *p* < .05 was considered significant and is marked in boldface.

^a^ Missing data: 48 (3.5%). ^b^Missing data: 8 (0.6%).

**Table 2.** Factors Associated With Short Sleep Duration (<7 hr).

	Wald Value	Odds Ratio	95% CI	*p* Value
Sex (boy)	3.3	.70	[0.47, 1.03]	.071
Age (14–15 years)	7.7	3.7	[1.47, 9.30]	**.005**
Poor economic situation	5.0	1.7	[1.07, 2.66]	**.025**
Overweight/obesity	7.4	1.9	[1.20, 3.08]	**.006**
School failure (≥1 failed subject)	8.8	2.1	[1.28, 3.30]	**.003**

*Note*. *n* = 1,363. Hosmer and Lemeshow’s goodness-of-fit test, *p* = .802; Nagelkerke *R* ^2^ = .055; tolerance > 0.9. CI = confidence interval. *p* < .05 was considered significant and is marked in boldface.

The students also described whether they had failed any subjects during the previous school year (“Have you failed in a subject during the last year?”) with response options: 0, 1–2, and ≥3 subjects. In Table 2, the responses were dichotomized, so that no failed school subjects during the last year were coded 0, and all other response options 1. The sex (girl/boy) and age (birth year) of each participant were also reported. In Table 2, age was dichotomized, so that 13 years were coded 0 and ages 14 and 15 were coded 1.

The BMI was calculated by dividing each student’s weight (kg) by the square of their height (m). The school nurses measured the height and weight of the students while wearing light clothing without shoes. Height was measured on a manual height board to the nearest 0.1 cm, and weight was measured on a standard digital scale to the nearest 0.1 kg. Obesity was defined as a BMI exceeding 30, and overweight was defined as a BMI exceeding 25 on a curve with age- and sex-specific cutoff points corresponding to the definitions of overweight or obesity for age 18 (Cole et al., 2000; Daniels, 2009b). The BMI data were sorted into three groups: normal weight, overweight, and obesity. In Table 2, the BMI data were dichotomized, so that normal weight was coded 0 and overweight and obesity were coded 1.

### Statistical Analysis

The statistical analyses were performed using IBM SPSS Version 24. The data were first analyzed using descriptive statistics with frequencies and percentages. Chi-square tests were used to investigate the association between short sleep duration and the following independent variables: sex, age, perceived economic situation, school failure, and overweight/obesity. A short sleep duration was defined as <7 hr/night (Fuligni et al., 2018; Grandner et al., 2015; Koinis-Mitchell et al., 2017; Lee et al., 2016). A multiple logistic regression (enter method) was used to analyze the extent to which the independent variables could explain the dependent variable (sleep duration: ≥7 hr/night = 0, <7 hr/night = 1). A *p* value <.05 was considered statistically significant, and 95% confidence intervals were estimated (Creswell & Creswell, 2017).

## Results

All students at the schools (*n* = 20) in the included area were invited to participate. The response rate was 72.7% (*n* = 1,518). However, 131 (8.6%) participants were excluded for not providing the information required to calculate their BMI. The group that provided BMI-related information did not differ in gender, age, or sleeping habits from the group that did not (*p* > .05). An additional 24 participants did not respond to the questions regarding sleep duration. Therefore, the analysis is based on 1,363 participants.

The participants’ ages ranged from 13 to 15 years, with a mean (*SD*) age of 13.9 (0.4) years. Most participants (94.3%) reported their families were financially “average,” “rather well off,” or “very well off.” The prevalence of overweight/obesity was 14.7% among girls and 15.9% among boys, while the obesity rates were 3.5% among girls and 3.4% among boys (Table 1). A sleep duration of <7 hr was associated with overweight/obesity, school failure, a poorer perceived economic situation, and older age (Table 2). Whereas 7.7% of the students with normal weight slept <7 hr, 11.5% and 21.7% of the students with overweight and obesity slept <7 hr. A sleep duration <7 hr was found among 7.6% of the students without any failed subjects, compared with 12.9% of the students with one to two failed subjects, and 21.7% among students with at least three failed subjects. Although we found significant associations between reduced sleep and all independent variables but sex, the Nagelkerke test suggests these variables explained only a minority of the odds of having a sleep duration <7 hr.

## Discussion

The aim of this study was to investigate the association between sleep duration, overweight/obesity, and school failure among adolescents aged 13–15. The key findings were that sleeping <7 hr per night was associated with overweight/obesity, school failure, and a poor economic situation. Other studies from Sweden and the United States have also found associations between a short sleep duration and academic performance (Fulugni et al., 2018; Jakobsson et al., 2019; Titova et al., 2015; Wernette & Emory, 2017). Also, Titova et al. (2015) found that school failure was more common among students who slept <7–8 hr, and their study also found an association between sleep quality and school failure, especially among girls, that is, girls complaining of sleep problems are at risk for failing school subjects.

There is a need to support adolescents in promoting healthy sleep habits. While the adolescent brain is developing, a pervasive issue is acquiring sufficient sleep. Importantly, insufficient sleep is associated with attention deficits, a lack of concentration, reduced cognitive functioning, and risk-taking behaviors (Kryger et al., 2011). Continued sleep deficiency among adolescents is likely due to a combination of puberty changes, school and after-school commitments, and lifestyle habits, including the lack of a bedtime routine and technology usage (Short et al., 2019). For example, sending and receiving text messages at night has been found to be associated with reduced sleep and tiredness during the school day (Garmy & Ward, 2018; Garmy et al., 2019). Adolescents who went to bed when they were tired, rather than when they have finished their electronic media use (messaging/socializing) or when the TV show was over, went to bed earlier and had a longer sleep duration on school nights (Short et al., 2019). There is a need to encourage adolescents to use their bodily cues to indicate when it is time for bed, rather than relying on an external cue (Short et al., 2019).

Less than 7 hr of sleep was more common among students with a self-reported poor economic situation, compared with students who reported their family financial status as average or good in our study. Sleep problems among Danish students aged 11–15 were found to be more common among students in families with lower socioeconomic status (Holstein et al., 2019). We do not know the reasons for this, but a hypothesis is that more presleep worries shorten sleep duration and increase sleep problems.

Our observed association between obesity and <7 hr sleep replicates findings from other studies (Grandner et al., 2015; Koinis-Mitchell et al., 2017; Lee et al., 2016). However, Calmaro et al. (2010) did not find an association between short sleep (<6 hr) and obesity, suggesting that obesity has many different causes and mechanisms. Obesity among adolescents is a public health concern, and its prevention is urgent due to the health consequences (e.g., high blood pressure, diabetes, inflammation), as well as increased health-care costs (Aggarwal & Jain, 2018). In conjunction with healthy food habits and physical activity, adequate sleep and healthy sleep habits are important factors to consider in preventing obesity.

### Implications for School Nursing

The rationale for this study was to provide school nurses with empirical evidence about the associations between sleep duration, overweight/obesity, and school success. This evidence could aid school nurses in counseling adolescents and their parents. The prevention and reduction of obesity are important challenges in public health, and sleep habits could represent a modifiable lifestyle variable that is free and fairly simple to change. The parental enforcement of bedtime rules has been found to increase sleep duration on weeknights by 1 hr, and the enforcement of rules regarding watching TV or using smartphones at night has been shown to increase sleep duration by 0.6 and 0.9 hr, respectively (Buxton et al., 2015). Therefore, school nurses should encourage parents and guardians to discuss bedtimes and access to screens at night.

### Limitations

One limitation of this study is its cross-sectional design, which means that it cannot determine causal relationships (Creswell & Creswell, 2017). The dependent variable in this study was sleep duration, whereas obesity and academic success were independent variables. There is the possibility that obesity itself could result in sleep difficulties by causing obstructive sleep apnea, which can lead to poor sleep quality (Hamilton & Joosten, 2017). A large study has identified a variety of sleep predictors, including some that were not investigated in this study such as ethnicity, cigarette smoking, and alcohol consumption (Kruger et al., 2014). Another limitation is that we did not control for exercise and dietarian patterns. Considering these variables as covariates in future studies could yield a more detailed picture of how sleep quality and being overweight are associated.

Some participants did not agree to provide their height or weight, which made it impossible to calculate their BMI. We did not investigate why these individuals declined to have their height or weight measured. However, because the group that did provide their BMI-related data was similar in all other respects to the group that did not, we have assumed that the latter group’s BMI values were similar to those recorded.

We only asked about sleep duration on school nights and did not ask any questions regarding sleep quality, which may be important to consider in future studies. Another limitation is that the sleep duration was self-reported, which poses a risk of recall bias and social desirability.

## Conclusion

The results of this study indicate that sleep habits should be taken into consideration as a part of the prevention and treatment of overweight/obesity as well as for promoting school success. Sleeping <7 hr per night was shown to be linked with overweight/obesity and school failure. School nurses can play an important role in conveying information about the benefits of adequate sleep and its association with a healthy weight and school success to parents and adolescents alike.

## References

[bibr1-1059840520901335] Aggarwal B. Jain V. (2018). Obesity in children: Definition, etiology and approach. Indian Journal of Pediatrics, 85, 463–471. 10.1007/s12098-017-2531-x 29177599

[bibr2-1059840520901335] Basch C. E. Basch C. H. Ruggles K. V. Rajan S. (2014). Prevalence of sleep duration on an average school night among 4 nationally representative successive samples of American high school students, 2007–2013. Preventing Chronic Disease, 11, E216 10.5888/pcd11.140383 25496556 PMC4264412

[bibr3-1059840520901335] Boeke C. E. Storfer-Isser A. Redline S. Taveras E. M. (2014). Childhood sleep duration and quality in relation to leptin concentration in two cohort studies. Sleep, 37, 613–620. 10.5665/sleep.3510 24587585 PMC3920328

[bibr4-1059840520901335] Buxton O. M. Chang A.-M. Spilsbury J. C. Bos T. Emsellem H. Knutson K. L. (2015). Sleep in the modern family: Protective family routines for child and adolescent sleep. Sleep Health, 1, 15–27. 26779564 10.1016/j.sleh.2014.12.002 PMC4712736

[bibr5-1059840520901335] Buzek T. Poulain T. Vogel M. Engel C. Bussler S. Korner A. Hiemisch A. Kiess W. (2019). Relations between sleep duration with overweight and academic stress—Just a matter of the socioeconomic status? Sleep Health, 5, 208–215. 10.1016/j.sleh.2018.12.004 30928123

[bibr6-1059840520901335] Calamaro C. J. Park S. Mason T. B. Marcus C. L. Weaver T. E. Pack A. Ratcliffe S. J. (2010). Shortened sleep duration does not predict obesity in adolescents. Journal of Sleep Research, 19, 559–566. 10.1111/j.1365-2869.2010.00840.x 20545836 PMC2965312

[bibr7-1059840520901335] Cole T. J. Bellizzi M. C. Flegal K. M. Dietz W. H. (2000). Establishing a standard definition for child overweight and obesity worldwide: International survey. British Medical Journal, 320, 1240 10.1136/bmj.320.7244.1240 10797032 PMC27365

[bibr8-1059840520901335] Creswell J. W. Creswell J. D. (2017). Research design: Qualitative, quantitative, and mixed methods approaches. Sage.

[bibr9-1059840520901335] Daniels S. R. (2009a). Complications of obesity in children and adolescents. International Journal of Obesity, 33, S60–S65. 10.1038/ijo.2009.20 19363511

[bibr10-1059840520901335] Daniels S. R. (2009b). The use of BMI in the clinical setting. Pediatrics, 124, S35–S41. 10.1542/peds.2008-3586F 19720666

[bibr11-1059840520901335] Eriksson M. Lingfors H. Golsater M. (2018). Trends in prevalence of thinness, overweight and obesity among Swedish children and adolescents between 2004 and 2015. Acta Paediatrica, 107, 1818–1825. 10.1111/apa.14356 29637596

[bibr12-1059840520901335] Fatima Y. Doi S. A. Mamun A. A. (2016). Sleep quality and obesity in young subjects: A meta-analysis. Obesity Reviews, 17, 1154–1166. 10.1111/obr.12444 27417913

[bibr13-1059840520901335] Felso R. Lohner S. Hollody K. Erhardt E. Molnar D. (2017). Relationship between sleep duration and childhood obesity: Systematic review including the potential underlying mechanisms. Nutrition, Metabolism, and Cardiovascular Diseases, 27, 751–761. 10.1016/j.numecd.2017.07.008 28818457

[bibr14-1059840520901335] Fuligni A. J. Arruda E. H. Krull J. L. Gonzales N. A. (2018). Adolescent sleep duration, variability, and peak levels of achievement and mental health. Child Development, 89, e18–e28. 10.1111/cdev.12729 28129442 PMC5529284

[bibr15-1059840520901335] Garmy P. Idecrans T. Hertz M. Sollerhed A. C. Hagell P. (2019). Is sleep duration associated with self-reported overall health, screen time, and nighttime texting among adolescents? Journal of International Medical Research. 10.1177/0300060519892399 PMC7782954 31849255

[bibr16-1059840520901335] Garmy P. Jakobsson U. Nyberg P. (2012). Development and psychometric evaluation of a new instrument for measuring sleep length and television and computer habits of Swedish school-age children. Journal of School Nursing, 28, 138–143. 10.1177/1059840511420878 21878574 PMC3697902

[bibr17-1059840520901335] Garmy P. Ward T. M. (2018). Sleep habits and nighttime texting among adolescents. Journal of School Nursing, 34, 121–127. 10.1177/1059840517704964 28421911

[bibr18-1059840520901335] Grandner M. A. Schopfer E. A. Sands-Lincoln M. Jackson N. Malhotra A. (2015). Relationship between sleep duration and body mass index depends on age. Obesity, 23, 2491–2498. 10.1002/oby.21247 26727118 PMC4700549

[bibr19-1059840520901335] Hamilton G. S. Joosten S. A. (2017). Obstructive sleep apnea and obesity. Australian Family Physician, 46, 460. 28697288

[bibr20-1059840520901335] Hirotsu C. Tufik S. Andersen M. L. (2015). Interactions between sleep, stress, and metabolism: From physiological to pathological conditions. Sleep Science, 8, 143–152. 26779321 10.1016/j.slsci.2015.09.002 PMC4688585

[bibr21-1059840520901335] Hirshkowitz M. Whiton K. Albert S. M. Alessi C. Bruni O. DonCarlos L. Hazen N. Herman J. Katz E. S. Kheirandish-Gozal L. Neubauer D. N. O’Donnell A. E. Ohayon M. Peever J. Rawding R. Sachdeva R. C. Setters B. Vitiello M. V. Ware J. C. Kheirandish-Gozal L. (2015). National Sleep Foundation’s sleep time duration recommendations: Methodology and results summary. Sleep Health, 1, 40–43. 29073412 10.1016/j.sleh.2014.12.010

[bibr22-1059840520901335] Holstein B. E. Ammitzboll J. Damsgaard M. T. Pant S. W. Pedersen T. P. Skovgaard A. M. (2019). Difficulties falling asleep among adolescents: Social inequality and time trends 1991-2018. Journal of Sleep Research, e12941 10.1111/jsr.12941 31692162

[bibr23-1059840520901335] Jakobsson M. Josefsson K. Jutengren G. Sandsjo L. Hogberg K. (2019). Sleep duration and sleeping difficulties among adolescents: Exploring associations with school stress, self-perception and technology use. Scandinavian Journal of Caring Sciences, 33, 197–206. 10.1111/scs.12621 30311255

[bibr24-1059840520901335] Kelly Y. Patalay P. Montgomery S. Sacker A. (2016). BMI development and early adolescent psychosocial well-being: UK millennium cohort study. Pediatrics, 138, e20160967 10.1542/peds.2016-0967 27940679 PMC5127062

[bibr25-1059840520901335] Koinis-Mitchell D. Rosario-Matos N. Ramírez R. R. García P. Canino G. J. Ortega A. N. (2017). Sleep, depressive/anxiety disorders, and obesity in Puerto Rican youth. Journal of Clinical Psychology in Medical Settings, 24, 59–73. 10.1007/s10880-017-9483-1 28239743 PMC5514541

[bibr26-1059840520901335] Kruger A. K. Reither E. N. Peppard P. E. Krueger P. M. Hale L. (2014). Do sleep-deprived adolescents make less-healthy food choices? British Journal of Nutrition, 111, 1898–1904. 24524288 10.1017/S0007114514000130 PMC4454607

[bibr27-1059840520901335] Kryger M. Roth G. Eloni D. Dement W. (2011). Principles and practice of sleep medicine. Elsevier.

[bibr28-1059840520901335] Kumar S. Kelly A. S. (2017). Review of childhood obesity: From epidemiology, etiology, and comorbidities to clinical assessment and treatment. Mayo Clinic Proceedings, 92, 251–265. 10.1016/j.mayocp.2016.09.017 28065514

[bibr29-1059840520901335] Lee B. H. Kang S. G. Choi J. W. Lee Y. J. (2016). The association between self-reported sleep duration and body mass index among Korean adolescents. Journal of Korean Medical Science, 31, 1996–2001. 10.3346/jkms.2016.31.12.1996 27822941 PMC5102866

[bibr30-1059840520901335] Leproult R. Van Cauter E. (2010). Role of sleep and sleep loss in hormonal release and metabolism. Endocrine Development, 17, 11–21. 10.1159/000262524 19955752 PMC3065172

[bibr31-1059840520901335] Lobstein T. Jackson-Leach R. Moodie M. L. Hall K. D. Gortmaker S. L. Swinburn B. A. James W. P. Wang Y. McPherson K. (2015). Child and adolescent obesity: Part of a bigger picture. Lancet, 385, 2510–2520. 10.1016/s0140-6736(14)61746-3 25703114 PMC4594797

[bibr32-1059840520901335] Matricciani L. Olds T. Petkov J. (2012). In search of lost sleep: Secular trends in the sleep time of school-aged children and adolescents. Sleep Medicine Reviews, 16, 203–211. 10.1016/j.smrv.2011.03.005 21612957

[bibr33-1059840520901335] Miller A. L. Lumeng J. C. LeBourgeois M. K. (2015). Sleep patterns and obesity in childhood. Current Opinion in Endocrinology, Diabetes, and Obesity, 22, 41–47. 10.1097/med.0000000000000125 PMC4437224 25517022

[bibr34-1059840520901335] Short M. A. Kuula L. Gradisar M. Pesonen A. K. (2019). How internal and external cues for bedtime affect sleep and adaptive functioning in adolescents. Sleep Med, 59, 1–6. 10.1016/j.sleep.2018.11.018 31150946

[bibr35-1059840520901335] Silva G. E. Goodwin J. L. Parthasarathy S. Sherrill D. L. Vana K. D. Drescher A. A. Quan S. F. (2011). Longitudinal association between short sleep, body weight, and emotional and learning problems in Hispanic and Caucasian children. Sleep, 34, 1197–1205. 10.5665/sleep.1238 21886357 PMC3157661

[bibr36-1059840520901335] Taveras E. M. Rifas-Shiman S. L. Oken E. Gunderson E. P. Gillman M. W. (2008). Short sleep duration in infancy and risk of childhood overweight. Archives of Pediatrics & Adolescent Medicine, 162, 305–311. 10.1001/archpedi.162.4.305 18391138 PMC2650815

[bibr37-1059840520901335] Titova O. E. Hogenkamp P. S. Jacobsson J. A. Feldman I. Schioth H. B. Benedict C. (2015). Associations of self-reported sleep disturbance and duration with academic failure in community-dwelling Swedish adolescents: Sleep and academic performance at school. Sleep Medicine, 16, 87–93. 10.1016/j.sleep.2014.09.004 25441744

[bibr38-1059840520901335] Wernette M. J. Emory J. (2017). Student bedtimes, academic performance, and health in a residential high school. Journal of School Nursing, 33, 264–268. 10.1177/1059840516677323 28726585

[bibr39-1059840520901335] Wolfson A. R. Carskadon M. A. Acebo C. Seifer R. Fallone G. Labyak S. E. Martin J. L. (2003). Evidence for the validity of a sleep habits survey for adolescents. Sleep, 26, 213–216. 12683482 10.1093/sleep/26.2.213

[bibr40-1059840520901335] World Health Organization. (2017). Report of the commission on ending childhood obesity: Implementation plan: Executive summary (No. WHO/NMH/PND/ECHO/17.1) World Health Organization.

[bibr41-1059840520901335] Yanovski J. A. (2015). Pediatric obesity. An introduction. Appetite, 93, 3–12. 10.1016/j.appet.2015.03.028 25836737 PMC4546881

